# Antibiotic prescribing in UK out-of-hours primary care services: a realist-informed scoping review of training and guidelines for healthcare professionals

**DOI:** 10.3399/BJGPO.2020.0167

**Published:** 2021-04-28

**Authors:** Paula Gomes Alves, Gail Hayward, Geraldine Leydon, Rebecca Barnes, Catherine Woods, Joseph Webb, Matthew Booker, Helen Ireton, Sue Latter, Paul Little, Michael Moore, Clare-Louise Nicholls, Fiona Stevenson

**Affiliations:** 1 University of Greenwich, London, UK; 2 University of Oxford, Oxford, UK; 3 University of Southampton, Southampton, UK; 4 University of Bristol, Bristol, UK; 5 Registered Nurse, Solent NHS Trust, Portsmouth, UK; 6 Registered Nurse, BrisDoc Heathcare Services, Bristol, UK; 7 University College London, London, UK

**Keywords:** prescribing, guidelines, community care, anti-bacterial agents, after-hours care, drug resistance, bacterial

## Abstract

**Background:**

Antibiotic overuse has contributed to antimicrobial resistance, which is a global public health problem. In the UK, despite the fall in rates of antibiotic prescription since 2013, prescribing levels remain high in comparison with other European countries. Prescribing in out-of-hours (OOH) care provides unique challenges for prudent prescribing, for which professionals may not be prepared.

**Aim:**

To explore the guidance available to professionals on prescribing antibiotics for common infections in OOH primary care within the UK, with a focus on training resources, guidelines, and clinical recommendations.

**Design & setting:**

A realist-informed scoping review of peer-reviewed articles and grey literature.

**Method:**

The review focused on antibiotic prescribing OOH (for example, clinical guidelines and training videos). General prescribing guidance was searched whenever OOH-focused resources were unavailable. Electronic databases and websites of national agencies and professional societies were searched following Preferred Reporting Items for Systematic Reviews and Meta-Analyses (PRISMA) standards. Findings were organised according to realist review components, that is, mechanisms, contexts, and outcomes.

**Results:**

In total, 46 clinical guidelines and eight training resources were identified. Clinical guidelines targeted adults and children, and included recommendations on prescription strategy, spectrum of the antibiotic prescribed, communication with patients, treatment duration, and decision-making processes. No clinical guidelines or training resources focusing specifically on OOH were found.

**Conclusion:**

The results highlight a lack of knowledge about whether existing resources address the challenges faced by OOH antibiotic prescribers. Further research is needed to explore the training needs of OOH health professionals, and whether further OOH-focused resources need to be developed given the rates of antibiotic prescribing in this setting.

## How this fit in

A higher proportion of antibiotics are prescribed in OOH primary care services, which may contribute to antibiotic resistance. Prescribing OOH has additional challenges for health professionals, including managing patient anxiety and professionals being unfamiliar with patients’ prior medical history and social circumstances. There is a lack of specific guidance for health professionals prescribing antibiotics in OOH primary care services. Providing evidence-based training targeting health professionals working OOH may increase prudent antibiotic prescribing.

## Introduction

Overuse and inappropriate prescribing of antibiotics is a worldwide public health concern as a major driver of antibiotic resistance.^1^ In the UK, 81% of all antibiotic prescriptions in 2017 were in primary care,^2^ including general practice and other community settings such as OOH services. Although antibiotic prescribing rates in general practice in the UK have fallen since 2013, rates of prescribing in OOH settings are increasing.^3^ Approximately, 15% of OOH consultations result in an antibiotic prescription, with suggestions this could represent a partial shift of antibiotic prescribing from in-hours to OOH primary care.^4^ Prescribing of antibiotics OOH in the UK is high in relation to other European countries such as the Netherlands.^5^ Moreover, a substantial proportion of UK prescriptions have been reported as not clinically warranted,^6,7^ with research indicating that if the level of prescribing in the UK decreased there would not be an increase in complications.^8^

Providing training in health care is key to improving quality of care and reducing prescription rates.^9^ In 2013, a multinational European study (which included the UK), showed that internet-based communication training plus C-reactive protein testing in general practice could reduce antibiotic prescribing for respiratory tract infections (RTIs) by 15%.^10^ In another study, communication skills training reduced antibiotic prescribing in 27% of cases.^11^ More recently, in a study conducted in Belgium, antibiotic prescriptions for RTIs dropped by 12% following online training on prudent antibiotic use and prescribing;^12^ and in the US, a clinical trial demonstrated that providing professionals with training on communication strategies and individualised prescribing feedback, decreased antibiotic prescribing for RTIs by 11% and produced a sustained reduction of 7% at follow-up.^13^

However, little is known about how existing clinical guidelines and training materials account for the unique challenges of prescribing OOH, which include professionals being unfamiliar with the patient’s medical history, the urgency of complaints, and patient anxiety and expectations.^14^ The goal of this study was to explore the guidance available to health professionals on prescribing antibiotics for common infections in OOH primary care within the UK. The review aimed to identify research evidence, training resources, guidelines, and clinical recommendations relating to antibiotic prescribing in OOH primary care in the UK. General antibiotic prescribing guidance was searched whenever OOH-focused guidance was unavailable.

## Method

The scoping review followed PRISMA guidelines.^15^ The review was part of a larger mixed-methods observational study exploring how professionals and patients (and/or caregivers) communicate about common infections and their treatment OOH (the OPEN Project; https://www.spcr.nihr.ac.uk/projects/414-understanding-antibiotic-prescribing-patterns-in-out-of-hours-ooh-primary-care).

For this review, the authors wanted to go beyond a traditional systematic review, which focuses on whether an intervention works or not, to instead explore the mechanisms underlying antibiotic prescribing in OOH and how antibiotics are prescribed in this setting. To understand these mechanisms, a realist review framework was employed. Realist reviews are exploratory by nature and aim to develop theories about the process that make interventions work by identifying *'* *mechanisms, contexts* *,* *and outcomes* *'* of complex scenarios.^16^ Although theory generating was beyond the scope of this article, this framework guided the data synthesis towards the identification of the mechanisms underlying antibiotic prescribing.

### Eligibility criteria

The authors were interested in peer-reviewed articles and grey literature concerned with how primary care professionals are trained and guided on how to prescribe antibiotics OOH in the UK. Training materials, clinical guidelines, reports and audits, and research articles were included. To be eligible for the review, these materials had to: be developed for the UK context or used in the UK; contain information relevant to patients of all ages and the general population; and refer to common conditions treated in primary care (see Table 1 for full inclusion and exclusion criteria). Preliminary searches (as described in the next section) identified peer-reviewed articles about antibiotic prescribing OOH, but no guidelines and training materials designed specifically for OOH. For this reason, the search was expanded to include any guidelines for antibiotic prescribing in primary care (either in hours or OOH).

**Table 1.** Inclusion and exclusion criteria

**Inclusion**	**Exclusion**
Antibiotic prescribing in primary care	Antibiotic prescribing in secondary, tertiary care services, and dentistry
Child, adolescent, and adult health	Languages other than English
Male and female patients	Published before 2008
Respiratory, ear, skin, urinary tract infections, and sepsis	All other infections
Developed and/or used in UK	

### Information sources

Websites of major healthcare agencies (for example, Public Health England), websites of relevant professional associations (for example, Royal College of General Practitioners), and electronic databases (for example, MEDLINE) were searched (see Supplementary Table S1 for full list of sources used). Sources were identified based on an exploratory search of the literature, and research team and collaborators’ experience.

### Search strategy

The terminology used to perform the searches was informed by preliminary readings of key studies about antibiotic prescribing, as well as consultations with experts and local provider organisations (Table 2).

**Table 2.** Key search terms

**Type of document**	**Search term**	**Alternative search terms**	**Associated subjective headings (MeSH terms, exploded**)
Peer-reviewed articles only	Out of hours	Urgent and emergency care After hours Accident and emergency service	After-hours care Emergency medicine
Peer-reviewed articles Other documents (clinical guidelines and training materials)	Antibiotic	Antibacterial Antimicrobial	Antibacterial agents
	Prescription guidelines	Guidance on prescribing Guidance for dispensing Good practice in prescribing	Drug prescriptions Practice guidelines as topic Drug utilisation Practice patterns, physicians’ Guideline adherence
	Professional training	Professional development Professional skills Continuing professional development	Education, medical Education, nursing

The search process was conducted iteratively as follows: an initial search strategy using a preliminary list of keywords was devised with librarians at the Royal Free NHS Hospital (London); a trial search was conducted to pilot the accuracy and relevance of those keywords; the list of keywords was adjusted until a final list was obtained; and finally, a second search was run where all the keywords were used in all sources and databases (see Supplementary Table S2 to access the full search strategy used). The searches were conducted in two stages: between July and October 2018 and then updated in March 2020, using the same sources and keywords.

### Selection of sources of evidence

The search results were saved and managed using EndNote (version 9). Documents were initially screened for inclusion based on title and abstract (if any) and table of contents or index. Whenever necessary the full text was consulted. All documents that met the inclusion criteria were selected. One author led the selection process with two other authors leading an independent 10% reference review for quality assurance.

The preliminary list of selected documents was circulated to the full research team and collaborators to ensure all key references were included, which resulted in the identification of additional documents. This list was also shared with two OOH service providers (OPEN Project partners) so that local guidelines and training materials could be identified and included in the search. The search ended in April 2020.

The heterogeneity of document formats included for review hindered the use of standardised and structured quality appraisal checklists. However, the relevance and quality of the documents considered for inclusion was discussed and agreed within the research team, which included healthcare researchers and primary healthcare practitioners.

### Data extraction and synthesis

The process began by reading the full text of selected documents. Descriptive data, such as target population and year of publication, were compiled in a data extraction form, which served as a *'* *template to interrogate the papers* *'*.^17^ Data extraction was done iteratively, with new categories being included whenever required.

The data synthesis aimed to consolidate the authors' understanding of what is known about training and clinical recommendations for antibiotic prescribing in OOH primary care services. Data were synthesised as follows:

#### Contexts

Research articles and clinical guidelines provided information about where healthcare professionals can find training and clinical guidelines for antibiotic prescribing. For this, the information regarding the year of publication, country, setting, study design, clinical condition, and clinical population was summarised for each document, and a descriptive analysis of these data was conducted, based on counts and frequencies.

#### Mechanisms

Clinical guidelines provided information about the type of recommendations (mechanisms) healthcare professionals are provided with to prescribe antibiotics OOH. For this, verbatim quotations were extracted from guidelines and these data were synthesised using a qualitative meta synthesis approach:^18^

1. gaining familiarity with data by actively reading each document, which meant appraising and extracting the relevant information;
2. coding the information, which represented key principles for antibiotic prescribing; and
3. grouping the final codes into themes and organisation of themes into a hierarchical structure.

#### Outcomes

Research articles provided information about the outcomes of studies in which health professionals were provided with training or clinical guidelines about how to prescribe antibiotics OOH. The results of each study were summarised and the key findings synthesised.

## Results

### Search results

**Figure 1.**
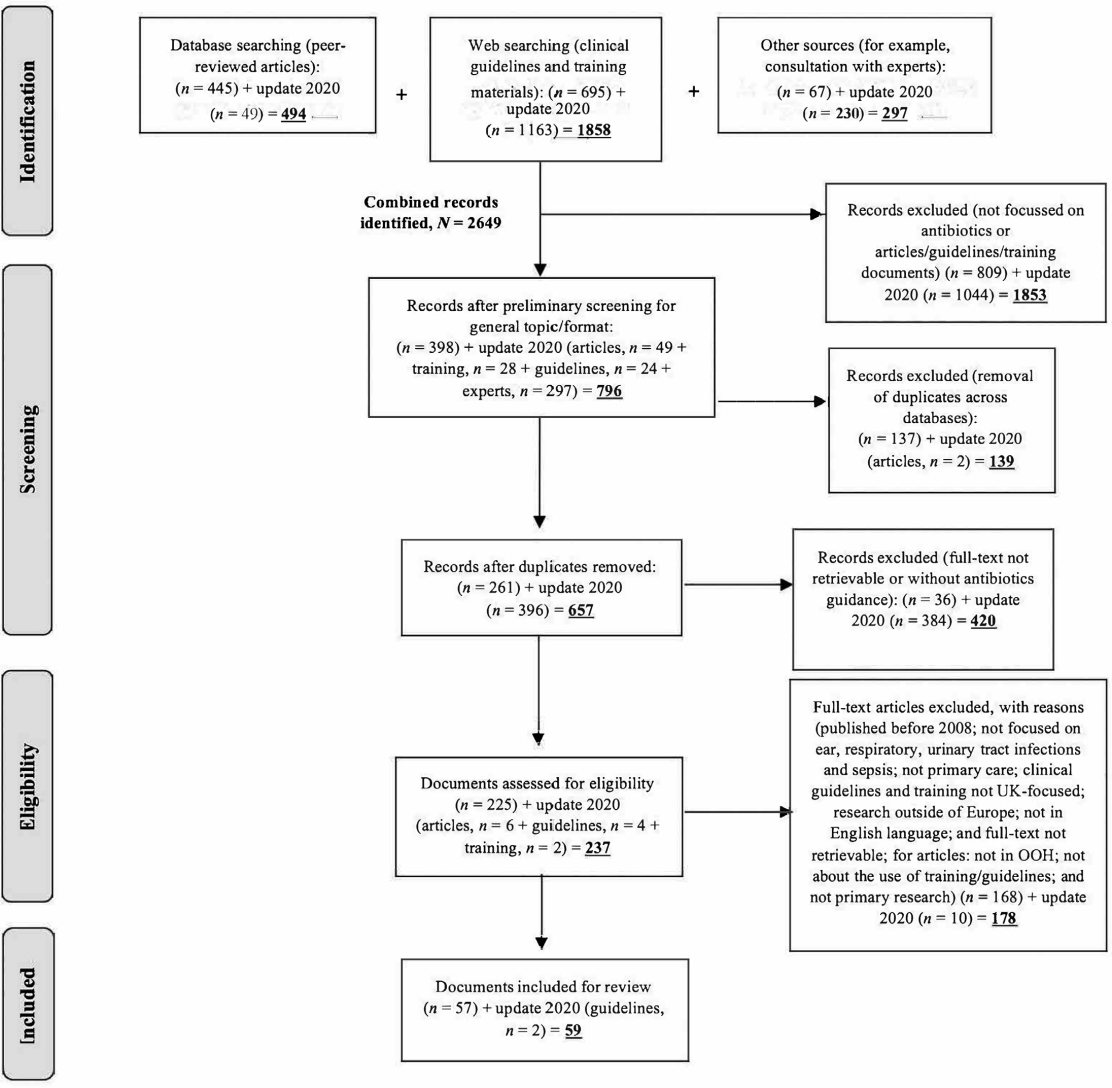
Selection flowchart and reasons for exclusion

The searches yielded a combined total of 2649 documents potentially eligible for review. After removing duplicates and preliminary screenings, 657 (25%) were included in the full-text screening. Of these, 59 (2% of initial yield) were included for review (see Figure 1). This comprised: five peer-reviewed articles, 46 clinical guidelines, and eight sets of training materials (webinars and eLearning modules). The research team agreed that all selected documents were relevant and of sufficient quality to inform the topic under review.

### What is addressed in guidance for antibiotic prescribing?

After screening, five peer-reviewed articles were identified, of which two targeted only children and three targeted both adult and child patients. All five articles focused on in-hours care and GPs, but one also included pharmacists and a further one included nurses. Two of the articles focused on fever and one on RTIs. Two articles did not specify any health condition (see Supplementary Table S3).

Guidelines and training materials were not specific to OOH, but instead included guidance available to all prescribers in general practices and hospitals (see Supplementary Table S4). Guidelines fell into three categories: adults, children, and generic. Most guidelines were either non-specific or about RTIs, urinary tract infections, or sepsis. Most guidance targeted the UK only, with the remainders being international but used in the UK.

### Which clinical recommendations for antibiotic prescribing are being provided to primary care professionals?

Although not specific to OOH, clinical guidelines and training materials included a wide range of general recommendations for antibiotic prescribing (see Supplementary Table S4). The themes covered in these documents were grouped as follows: 1) spectrum of the antibiotic prescribed, that is, range of bacterial types that the antibiotic affects, including broad and narrow spectrum; 2) strategy to prescribe antibiotics, that is, immediate, no antibiotic, and delayed or ‘just in case’; 3) communication with patients, that is, information and advice about antibiotics; 4) treatment duration, that is, length of prescribed antibiotic course; 5) clinical benchmarking, that is, comparisons using clinical data to support and implement best practice; and 6) decision-making process, that is, steps involved in the process leading to antibiotic prescribing, including a no antibiotic decision.

The recommendations found across the literature were consensual, and no conflicting principles were identified. Moreover, half of the clinical recommendations synthesised (see Supplementary Table S4) were endorsed by >1 organisation. Approximately one-third of the recommendations were published both in UK-only guidance and international documents. Just over 60% of recommendations were either revised or published in the past 2 years.

### How do training and guidelines impact on antibiotic prescribing OOH?

There was limited mention in the literature about the outcomes of training interventions on antibiotic prescribing in OOH. The articles identified indicated that prescribers working OOH may face uncertainties about diagnosis,^19^ and that guidelines specifically related to working in OOH would be helpful in relation to the decision-making process.^20^ The literature also suggested that GPs and prescribing nurses may be prescribing antibiotics in OOH settings according to different standards and guidelines, and would appreciate supervision, discussion with peers, or clinical audits.^21^ Prescribing is likely to be reduced when family physicians have access to interactive booklets to facilitate communication with patients,^22^ and peer education programmes promote greater adherence to clinical guidelines.^23^ See Supplementary Table S3 for further details concerning the findings of the studies.

## Discussion

### Summary

The goal of this study was to explore the existing clinical recommendations for primary care professionals working OOH, regarding how and when to prescribe antibiotics. A realist-informed review was conducted to provide an overview of the contexts, mechanisms, and outcomes of these recommendations, described both in guidelines available to primary care professionals and research in the field.

The key finding is that there is a paucity of information about how professionals are being trained to prescribe antibiotics specifically in OOH and whether further, specific training is required. For instance, guidelines that were reviewed highlight that professionals should discuss treatment plans, manage expectations, and inform patients about the consequences of antibiotics and self-limiting conditions. However, there is a dearth of evidence regarding the basis for these recommendations, how they are being implemented in OOH settings, or indeed how appropriate they are for this context, where healthcare professionals often have to diagnose infection without access to laboratory results. Additionally, it is also clear that further empirical research is needed in this field, particularly in relation to whether training professionals in OOH contributes to lower prescribing rates.

### Strengths and limitations

The primary limitation of this review was the lack of information about training materials and guidelines focusing on OOH contexts. The search results were shared with two OOH service providers in the hopes of identifying further resources; however, no additional materials were identified. This made it difficult to produce definitive conclusions, aside from that professionals in OOH may not have access to specific training related to working in this context. Future studies should aim to engage with further OOH providers to ensure that local guidance is also considered. Nevertheless, the realist-informed review allowed the mechanisms underlying antibiotic prescribing in OOH to be considered, which may provide a platform on which to build future theoretical work. Another limitation is that owing to the heterogeneity of sources, quality assurance review was dependent on the expertise and judgement of the research team, as opposed to traditional standardised quality appraisal procedures.

### Comparison with existing literature

The limited research on prescribing antibiotics in OOH settings makes it difficult to compare the findings with previous literature. Previous studies have shown that educational interventions and training are likely to optimise prescribing competency^24^ and reduce inappropriate prescribing.^25^ The literature also indicates that general training for GPs and other clinicians working OOH could be expanded and standardised at national level. For instance, in a survey with over 1000 GP trainees in England, Hayward *et al* ^26^ found that only half of GP trainees received formal education sessions on OOH care, and no more than one-third were offered formal training in OOH home visits. Training in OOH care is generally perceived as a positive experience for GPs and trainees, suggesting that further training could be well received.^27^

### Implications for research and practice

This review highlights the need for further thought and discussion as to whether general clinical guidelines on antibiotic prescribing are adequately addressing the particularities and challenges of OOH care. This includes: 1) the management of patients by health professionals unfamiliar with their medical history; 2) limited access to medical records and testing; 3) higher levels of patient (and caregiver) pressure and patient anxiety; 4) perceived or actual urgency of complaints or acuity of infections on point; 5) patients who may have experienced difficulties accessing care and prescriptions in hours; and 6) little opportunity for patient follow-up.^14,21,28^ It is important to explore the needs of healthcare professionals working OOH and their preferences regarding the format and type of resources needed to support this work to ensure that the success of future interventions is optimised.

In terms of prescribing strategies, the review identified various recommendations for UK healthcare professionals when prescribing antibiotics. However, since none of these were tailored specifically to OOH care, it is uncertain whether the recommendations are relevant and feasible in this setting. For instance, research shows that delayed prescribing has been associated with a decrease in antibiotic use.^29–33^ Further research is needed to explore the benefits and barriers to offering delayed antibiotic prescriptions OOH where there may be particular patient expectations regarding the immediacy of the need for a prescription.

Moreover, as Colliers *et al* ^20^ suggest, based on primary care in Belgium, GPs working OOH do not always feel confident in their decision making regarding antibiotics and feel less responsible for these decisions owing to patients being 'unknown'. Similar findings were reported in the UK, where research highlighted the lack of prior relationship with patients as a factor hindering clinical decision making.^21^ On this basis, the authors believe that evidence-based training may help strengthen antibiotic stewardship practices in OOH contexts. Evidence-based targeted training may result in modifying OOH consulting behaviour and optimising prescribing practices in line with generalised antibiotic stewardship programmes, which have been shown to be effective in improving patient outcomes, producing savings for health services, and reducing antibiotic resistance.^33^

This review identified no current clinical guidelines or training resources focusing on antibiotic prescribing targeting professionals working in OOH primary care settings in the UK. In addition, it identified only limited research evidence about the use of educational interventions in this field. The review also suggests that even though they have been separated from in-hours GP services since 2004, it is unclear which guidelines are being used by medical and non-medical prescribers working OOH in the UK.

Further research is needed to ascertain whether the existing guidance is fit for the challenges faced in OOH settings, and whether and what other resources targeting antibiotic prescribing OOH need to be developed. Observational and exploratory research could be a first step towards understanding the context and training needs of professionals working OOH, and how training and updated guidelines could best support prescribing practices.
